# Staurosporine Increases Lentiviral Vector Transduction Efficiency of Human Hematopoietic Stem and Progenitor Cells

**DOI:** 10.1016/j.omtm.2018.04.001

**Published:** 2018-04-05

**Authors:** Gretchen Lewis, Lauryn Christiansen, Jessica McKenzie, Min Luo, Eli Pasackow, Yegor Smurnyy, Sean Harrington, Philip Gregory, Gabor Veres, Olivier Negre, Melissa Bonner

**Affiliations:** 1 bluebird bio, Inc., 60 Binney St., Cambridge, MA 02142, USA

**Keywords:** lentiviral, HSPC, transduction

## Abstract

Lentiviral vector (LVV)-mediated transduction of human CD34^+^ hematopoietic stem and progenitor cells (HSPCs) holds tremendous promise for the treatment of monogenic hematological diseases. This approach requires the generation of a sufficient proportion of gene-modified cells. We identified staurosporine, a serine/threonine kinase inhibitor, as a small molecule that could be added to the transduction process to increase the proportion of genetically modified HSPCs by overcoming a LVV entry barrier. Staurosporine increased vector copy number (VCN) approximately 2-fold when added to mobilized peripheral blood (mPB) CD34^+^ cells prior to transduction. Limited staurosporine treatment did not affect viability of cells post-transduction, and there was no difference in *in vitro* colony formation compared to vehicle-treated cells. Xenotransplantation studies identified a statistically significant increase in VCN in engrafted human cells in mouse bone marrow at 4 months post-transplantation compared to vehicle-treated cells. Prostaglandin E_2_ (PGE_2_) is known to increase transduction efficiency of HSPCs through a different mechanism. Combining staurosporine and PGE_2_ resulted in further enhancement of transduction efficiency, particularly in short-term HSPCs. The combinatorial use of small molecules, such as staurosporine and PGE_2_, to enhance LVV transduction of human CD34^+^ cells is a promising method to improve transduction efficiency and subsequent potential therapeutic benefit of gene therapy drug products.

## Introduction

Encouraging *ex vivo* gene therapy utilizing lentiviral vector (LVV) transduced human CD34^+^ hematopoietic stem and progenitor cells (HSPCs) has been documented in multiple diseases.^1^, ^2^, ^3^, ^4^, ^5^, ^6^ Even with numerous examples of promising clinical outcomes, transduction of long-term HSPCs (LT-HSPCs) remains challenging. Overcoming transduction barriers in LT-HSPCs, especially in indications where a high proportion of genetically modified cells is necessary for therapeutic benefit, is a significant focus of the field.^7^, ^8^, ^9^, ^10^, ^11^ To facilitate transgene delivery into LT-HSPCs, we and others have employed small molecules or peptides that can be added to the transduction process to overcome barriers preventing LVV transduction and thus to increase the proportion of transduced LT-HSPCs. Similar efforts have been undertaken by others and have led to the identification of rapamycin, cyclosporin, vectofusin, and prostaglandin E_2_ (PGE_2_) to increase LVV transduction efficiency in cells.^7^, ^8^, ^9^, ^10^, ^11^

Here, we investigated the potential of staurosporine, a serine/threonine kinase inhibitor, to enhance the transduction of LVVs in mobilized peripheral blood (mPB) CD34^+^ cells both *in vitro* and *in vivo* using a xenogeneic NOD-Cg-Prkdc^scid^Il2rg^tm1Wjl^/Sz (NSG) mouse model. Staurosporine treatment has been previously demonstrated to cause chromatin relaxation in metaphase cells and increase HIV-1 integration in metaphase-arrested cells.^12^ A short pre-treatment of refractory resting T cells with staurosporine led to activation of cofilin and an increase in actin depolymerization, which was shown to promote the nuclear localization of the viral pre-integration complex and resulted in an increase in integrated viral genomes.^13^ In a separate study, staurosporine treatment led to a 150% increase in HIV-1 infection, measured by p24, of CD4^+^ T cells.^14^ Although HIV infection and LVV transduction utilize different mechanisms to overcome the cellular membrane barrier, it has been shown that other methods used to increase transduction efficiency of LVV in HSPCs, such as spinoculation, which has been utilized to transduce HSPCs with both gammaretroviral vector and LVV, cause a similar activation of cortical actin dynamics, suggesting that this cellular membrane entry barrier might be a common restriction point for both HIV infection and LVV transduction.^15^, ^16^, ^17^, ^18^

In this study, we found that pre-treatment of CD34^+^ cells with staurosporine prior to transduction led to an approximate 2- to 3-fold increase in entry of LVV, as measured via the BlaM assay.^19^ Investigation into the mechanism revealed that staurosporine treatment inhibits cofilin phosphorylation at serine 3, which leads to increased actin dynamics, or treadmilling.^20^ We further show that when combined with PGE_2_, an entry-independent modulator of LVV transduction, we can increase LVV transduction efficiency further than with either compound used independently. The increased transduction efficiencies *in vitro* led to increased transduction of engrafted human cells in a xenotransplant NSG mouse model without adverse effects on engraftment or *in vivo* differentiation capabilities of the HSPCs.

## Results

### Staurosporine Treatment Increases Transduction of Human CD34^+^ Cells

**Figure 1:**
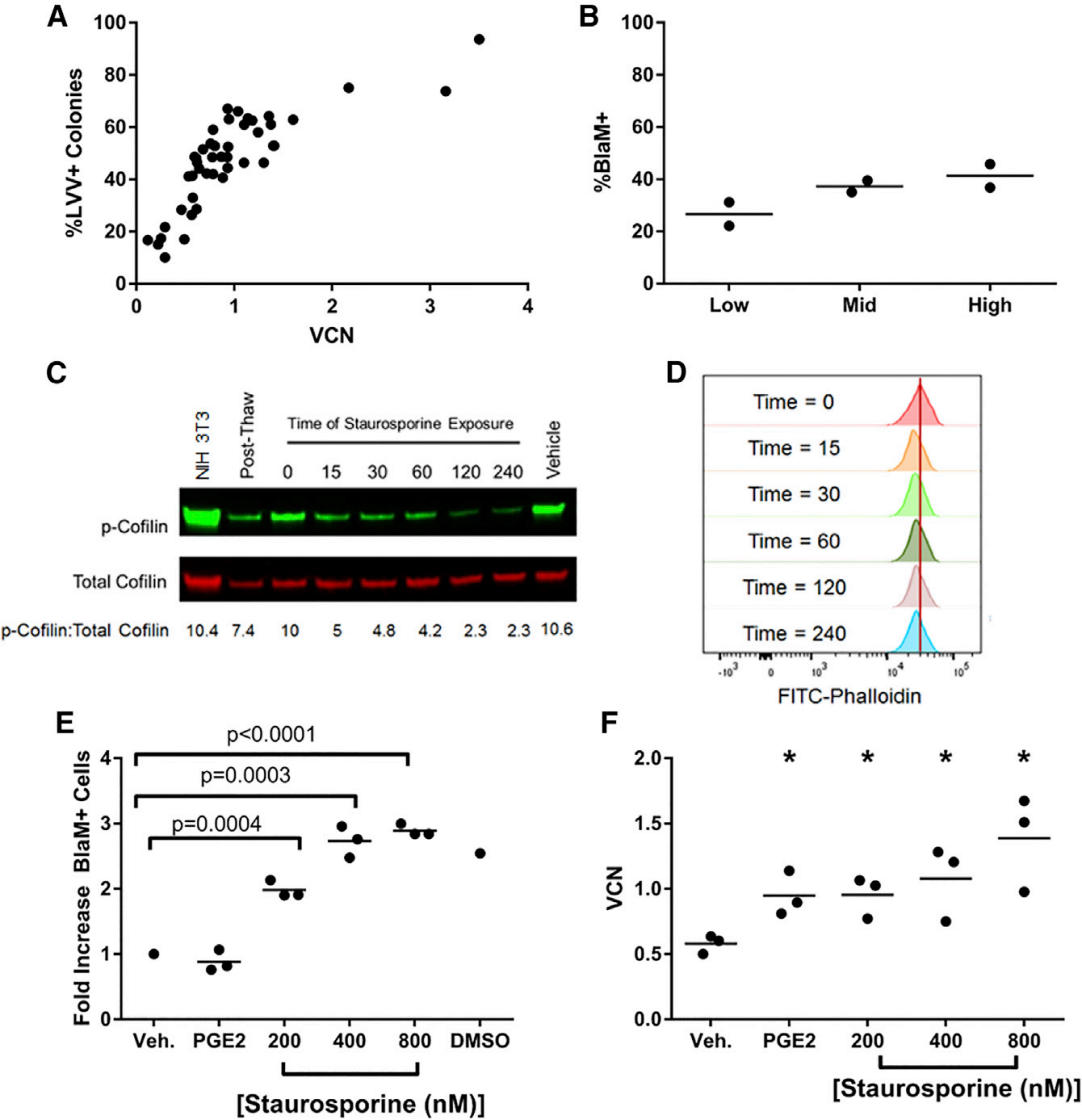
Staurosporine Treatment Increases Transduction of Human CD34^+^ Cells (A) Variability of transduction. Forty-five research-scale CD34^+^ transductions using 17 unique mPB cell lots and six different LVV lots. VCN (vector copies per diploid genome, c/dg) is from pooled colonies from methylcellulose. %LVV^+^ colonies are the proportion of vector-bearing colonies. (B) CD34^+^ cell lots identified via BB305 LVV research-scale transductions to be low (VCN < 0.5), mid (0.5 < VCN < 1), and high (VCN > 1) transducers were assessed for LVV entry using a LVV containing a BlaM-Vpr fusion protein. The proportion of cells containing LVV 2 hr post-transduction (% BlaM^+^) are shown. (C) Western blot analysis of CD34^+^ cells post-thaw or pre-stimulated and treated with 800 nM staurosporine for indicated time (in minutes). Vehicle indicates treatment with 0.1% DMSO for 2 hr. Ratios of band intensities of p-cofilin to total cofilin are listed below respective lanes. (D) Representative histograms of FITC-labeled phalloidin (F-actin) staining of CD34^+^ cells treated with 800 nM staurosporine for indicated time (in minutes). Red vertical bar drawn at MFI of untreated cells. (E) CD34^+^ cells were transduced for 2 hr with a BlaM-containing LVV, and BlaM activity was measured with the GeneBLAzer kit. (Veh, vehicle, 0.1% DMSO; PGE_2_, 10 μM PGE_2_ during transduction; staurosporine pre-treatment before transduction; DMSO, 5% DMSO during transduction). (F) CD34^+^ cells treated as in (E) and transduction carried out for 24 hr were plated in methylcellulose and pooled colonies were analyzed by qPCR for VCN. *p < 0.05.

Consistently achieving both high average vector copy numbers (VCNs) and a high proportion of transduced cells (%LVV^+^) in HSPCs is a challenge in the gene therapy field. Figure 1A shows the variability in HSPC transduction performed at research scale using CD34^+^ cells from more than 15 different donors and using six different LVV lots at clinically relevant MOIs. Of 45 research-scale transductions, only 33% achieved a VCN >1 and 44% contained greater than 50% modified cells. These data highlight the potential difficulty in manufacturing gene-modified HSPCs for therapeutic use in diseases where high expression of the therapeutic protein and/or a high proportion of modified cells is needed for efficacy. Representative cell lots characterized as low, mid, or high transducers based on research-scale transductions with BB305 LVV were analyzed for evidence of LVV entry into HSPCs via the BlaM assay.^19^, ^21^ There was a trend of increasing BlaM activity with increased innate achievable transduction level of cells (Figure 1B); however, it is not statistically significant (p = 0.15, one-way ANOVA). Even in cell lots capable of achieving high levels of transduction (defined as VCN > 1), less than 50% of the cells are BlaM^+^, indicating there is a barrier to LVV entry.

Staurosporine, a serine/threonine kinase inhibitor, has previously been shown to increase HIV infection of resting T cells by stimulating cortical actin dynamics.^13^ This is achieved by activation of cofilin, an integral protein for assembly and disassembly of filamentous actin, by preventing its inhibitory phosphorylation at serine 3 by LIMK1. To determine if cofilin phosphorylation and cortical actin dynamics could be potential targets for overcoming LVV transduction restriction in HSPCs, mPB CD34^+^ cells that had been pre-stimulated for 48 hr in cytokine-containing media were treated with 800 nM staurosporine for up to 4 hr and then analyzed for cofilin phosphorylation via western blot (Figure 1C) and filamentous actin staining with fluorescein isothiocyanate (FITC)-labeled phalloidin (Figure 1D). As can be seen in Figure 1C, there is a rapid decrease in the level of phosphorylated cofilin with staurosporine treatment with a maximum reduction reached by 2 hr of staurosporine exposure. Figure 1D shows a similar, rapid decrease in the mean fluorescence intensity (MFI) of filamentous actin staining with staurosporine treatment, and this stimulation in actin dynamics is also shown in more primitive CD34^+^CD38LoCD45RA-CD90^+^ cells (data not shown). Based on this data, we selected 2 hr of staurosporine treatment immediately prior to transduction to further evaluate effects on LVV transduction of HSPCs.

Human mPB CD34^+^ cells from healthy donors were cultured for approximately 48 hr in cytokine-containing media and then pre-treated for 2 hr with various concentrations of staurosporine or vehicle (0.1% DMSO) control. Staurosporine was then removed by media exchange and the cells were transduced with a BlaM-containing LVV in the presence of vehicle, 10 μM PGE_2_ (a recently identified transduction enhancer), or 5% DMSO as a positive control for LVV entry via cell membrane permeabilization.^10^, ^11^ Cells were analyzed 2 hr post-transduction for LVV entry using the GeneBLAzer kit. A 2- to 3-fold increase in LVV entry was observed when cells were pre-treated with increasing concentrations of staurosporine prior to transduction (Figure 1E). As previously shown, PGE_2_ had no effect on LVV entry.^11^ Cells similarly pre-treated with vehicle or staurosporine were transduced for 24 hr with LVV and then plated in methylcellulose. VCN analysis of pooled colonies demonstrated a statistically significant increase in VCN with either PGE_2_ treatment during transduction or a 2-hr pre-treatment with staurosporine compared to vehicle control (Figure 1F). Importantly, there was no difference in the viabilities of cells treated with 200 to 800 nM staurosporine for up to 2 hr, with all samples having >90% viable cells by trypan exclusion post-transduction and no difference in hematopoietic colony output in methylcellulose (Figure S1). However, a longer (24 hr) exposure of HSPCs to staurosporine resulted in decreased hematopoietic colony output in methylcellulose (Figure S1). Therefore, to avoid toxicity, it is important to limit the duration of staurosporine treatment.

**Figure 2:**
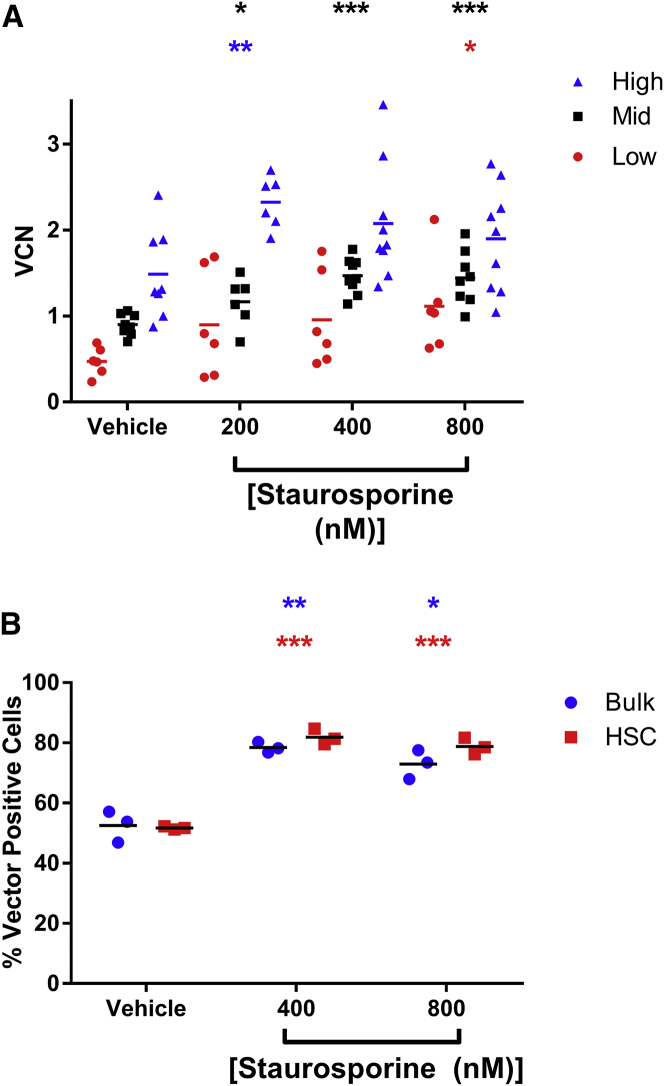
Staurosporine Treatment Increases Transduction in Multiple Cell Lots and in Phenotypically Defined LT-HSPCs (A) Six unique CD34^+^ cell lots previously identified as being low (VCN < 0.5), mid (0.5 < VCN < 1), and high (VCN > 1) transducers were treated with vehicle or indicated concentrations of staurosporine for 2 hr, washed, and transduced with BB305 LVV. Pooled colonies from methylcellulose were analyzed for VCN. Black asterisk is mid-transducer group compared to respective vehicle control. Blue asterisk is high-transducer group compared to respective vehicle control. Red asterisk is low-transducer group compared to respective vehicle control. (B) CD34^+^ cells were sorted into bulk (CD34^+^) and phenotypically defined LT-HSPCs (CD34^+^CD38LoCD90^+^CD45RA^−^). Phenotypic LT-HSPCs were CFSE labeled. Cells were cytokine cultured and then transduced with BB305 LVV following a 2-hr treatment with vehicle or staurosporine (400 or 800 nM). Cells were single-cell sorted CFSE-negative and CFSE-labeled 4 days post-transduction, and the percentage of vector-containing cells was assessed. Blue asterisk is bulk population relative to vehicle control. Red asterisk is HSC relative to vehicle control. *p < 0.05, **p < 0.005, ***p < 0.0005.

Achieving a high proportion of genetically modified cells is desirable, and in certain indications, the additional increase in VCN on a per-cell basis could also be beneficial, for example when high transgene expression is necessary for efficacy. To evaluate the potential for staurosporine treatment prior to transduction to result in higher VCNs, CD34^+^ cell lots characterized as low, mid, or high transducers were treated with different concentrations of staurosporine for 2 hr followed by transduction with BB305 LVV. Pooled methylcellulose colonies were evaluated for VCN. As can be seen in Figure 2A, there was a trend for increased VCN with increased concentration of staurosporine. Additionally, higher VCNs were consistently achieved in known high-transducing cell lots compared to low-transducing cell lots. Interestingly, the overall fold increase in VCN reveals an opposing trend where low-transducing cell lots are experiencing a higher fold increase in VCN with staurosporine treatment compared to high-transducing cell lots (Table S1). For example, on average the increase in low-transducing cell lots treated with 800 nM staurosporine is 2.4-fold, while in high-transducing cell lots the same staurosporine treatment results in a 1.3-fold increase in VCN. One possible explanation for this is that while staurosporine treatment facilitates LVV entry across all cell lots, non-entry-based intracellular LVV transduction restriction factors result in a plateau.^22^

Human CD34^+^ cells are a common starting material for HSPC-based gene therapy and LT-HSPCs represent a small fraction of the heterogenous CD34^+^ cell population. Any small molecule used to increase LVV transduction of bulk CD34^+^ cells needs simultaneously to increase the transduction of LT-HSPCs for any potential boost in efficacy of transduced cells to have a long-lasting effect. To investigate this, CD34^+^ cells were either bulk sorted or a specific HSPC sub-population (CD34^+^CD38LoCD90^+^CD45RA^−^) associated with true LT-HSPCs was isolated. The phenotypically defined LT-HSPC population was then carboxyfluorescein succinimidyl ester (CFSE)-labeled and recombined with bulk CD34^+^ cells to maintain standard transduction conditions of cell density and volume. Mixed cell populations were then either vehicle-treated or incubated with staurosporine for 2 hr prior to transduction with BB305 LVV. Four days post-transduction, either bulk or CFSE-labeled cells were single cell sorted into 96-well plates. Each well was then analyzed via nested qPCR for the presence of housekeeping gene RNaseP, indicating presence of a detectable cell in the well, and vector sequence, indicating the cell is transduced. The ratio between vector-containing wells and wells with detectable cells (%LVV^+^ cells) is illustrated in Figure 2B. The %LVV^+^ cells in both bulk CD34^+^ cells and phenotypic LT-HSPCs increased with staurosporine treatment compared to vehicle-treated cells, and there was no significant difference between the %LVV^+^ cells in bulk or phenotypically LT-HSPC populations. These data suggest that staurosporine treatment results in an increase in the proportion of genetically modified cells that would persist long term. Additionally, these data demonstrate that the increase in VCN seen with staurosporine treatment is at least partially due to an increase in the proportion of genetically modified cells and not solely due to an increase in the number of vector integrations per cell in the subpopulation of most permissive cells.

### Staurosporine Treatment Increases Transduction of Human Long-Term NSG Repopulating Cells

**Figure 3:**
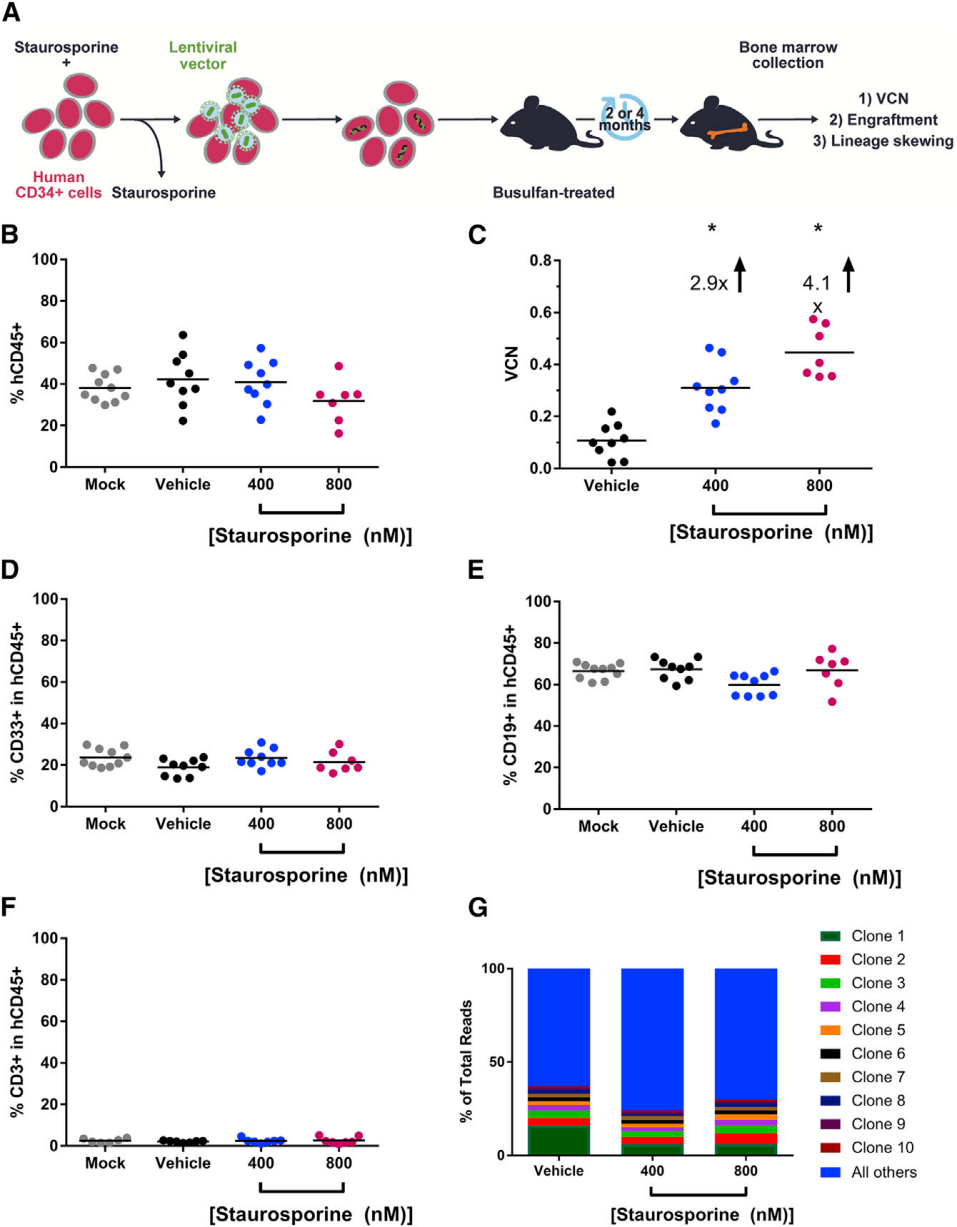
Staurosporine Increases the Proportion of Genetically Modified Long-Term Repopulating Cells (A) Schematic of NSG transplant. Cells were transduced after pre-treatment with vehicle or staurosporine (400 nM or 800 nM) and then transplanted via tail-vein injection into sub-lethally myeloablated NSG recipients. Four months post-transplantation, BM was analyzed for VCN and engraftment. (B) Engraftment of long-term repopulating human cells in NSG assessed by presence of human CD45. Each dot represents an individual mouse. (C) VCN of engrafted human cells in BM of NSG mice. (D) Assessment of *in vivo* myeloid differentiation of human cells (CD33^+^). (E) Assessment of *in vivo* B cell differentiation. (F) *In vivo* T cell differentiation. (G) Top 10 clones captured via integration site analysis (ISA) from BM of transplanted animals. Note that clones 1–10 represent different integration sites within each transplant arm. *p < 0.0001.

To further characterize the ability of staurosporine to increase the transduction of long-term repopulating cells, an NSG transplant was initiated, schematically represented in Figure 3A. Human CD34^+^ cells were transduced with BB305 LVV following a 2-hr treatment with vehicle, 400 nM, or 800 nM staurosporine. Immediately following transduction, cells were transplanted into busulfan-treated NSG mice. A subset of cells from each sample was put into culture for *in vitro* characterization (data not shown). Four months post-transplant, femurs were collected and the level of human CD45^+^ engraftment, the *in vivo* differentiation capabilities of long-term repopulating cells, and VCN were assessed. As can be seen in Figure 3B, there was no significant difference in the level of human chimerism as assessed by human CD45^+^ staining across all groups, indicating that the staurosporine treatment did not impact the long-term repopulating ability of the CD34^+^ cells. The VCN enhancement effect was statistically significant *in vivo* 4 months post-transplant (Figure 3C), with 400 nM staurosporine treatment leading to a 2.9-fold increase in VCN over vehicle-treated cells and 800 nM staurosporine treatment producing a 4.1-fold increase in VCN over vehicle-treated cells. Importantly, at this 4-month time point, we observed no difference in the ability of these cells to differentiate into either myeloid (Figure 3D), B or T lymphoid (Figures 3E and 3F) cells *in vivo*, suggesting that staurosporine treatment did not impact the lineage differentiation capacity of the engrafting CD34^+^ cells. Additionally, both the cells retained at time of transplant for *in vitro* analysis (Figure S2) and the engrafted cells (Figure 3G) represented a polyclonal population in all transduced arms, demonstrating that staurosporine treatment does not alter integration site profile nor confer bias to any clones. Additionally, integration sites were mapped within gene bodies to examine the integration profile with each treatment and staurosporine treatment did not skew the LVV integration profile of transduced cells (Figure S2).

### Combination Staurosporine and PGE_2_ Treatment Further Enhances Transduction of Human CD34^+^ Cells

**Figure 4:**
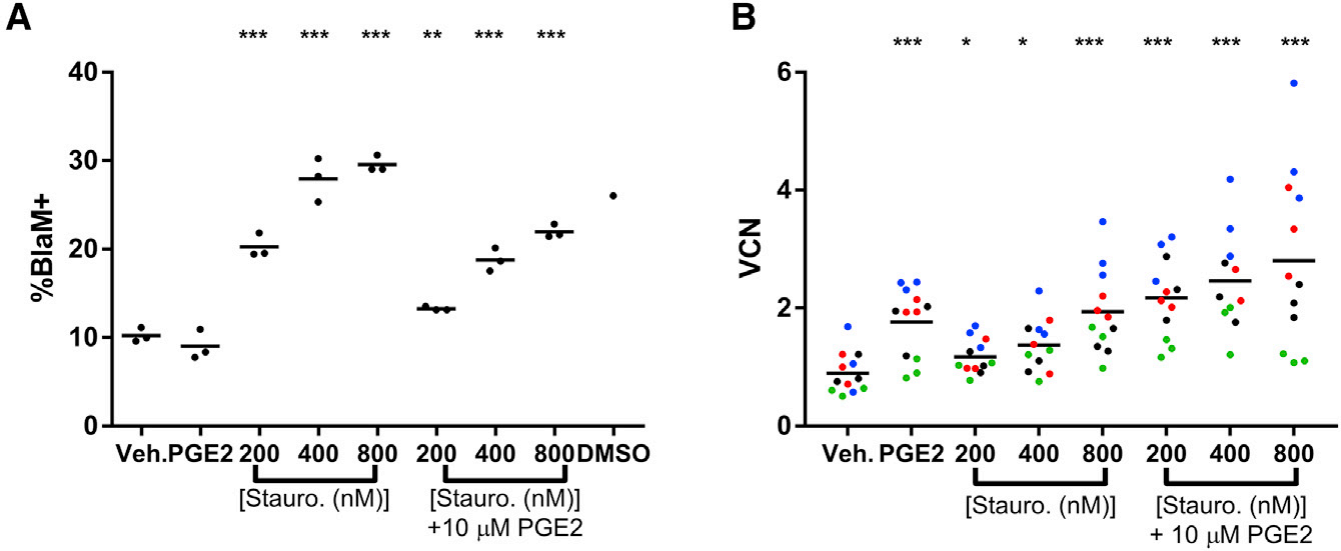
Combination Staurosporine and PGE_2_ Treatment Further Enhances Transduction of Human CD34^+^ Cells (A) CD34^+^ cells were treated for 2 hr with vehicle or staurosporine (200–800 nM) and then transduced with a BlaM-containing LVV for 2 hr in the presence of vehicle or 10 μM PGE_2_. Cells were then analyzed for presence of β-lactamase activity, indicating LVV entry. DMSO only = 5% DMSO as control for entry via cellular membrane permeabilization. (B) CD34^+^ cells were treated for 2 hr with vehicle or staurosporine (200–800 nM) and then transduced with a BlaM-containing LVV or BB305 LVV for 24 hr in the presence of vehicle or 10 μM PGE_2_. Pooled methylcellulose colonies were analyzed for VCN. Circles indicate individual replicates within an experiment; color indicates unique experiment. *p < 0.05, **p < 0.005, ***p < 0.0005, all compared to vehicle control.

We and others have demonstrated that addition of PGE_2_ to transduction media can increase the overall level of transduction of CD34^+^ cells.^10^, ^11^ Heffner et al.^11^ showed that this effect was elicited post-entry of LVV into the CD34^+^ cells, as PGE_2_ treatment did not impact LVV entry as determined by the BlaM assay (see also Figure 1E). Since staurosporine does increase LVV entry into CD34^+^ cells, we hypothesized that combining staurosporine pre-treatment and PGE_2_ addition to the transduction media could result in further enhancement of transduction by impacting transduction at distinct stages of the viral integration process. Human CD34^+^ cells were pre-stimulated with vehicle or staurosporine for 2 hr, transduced with a BlaM-containing LVV with the addition of vehicle or PGE_2_ for 2 hr, and then assessed for the proportion of cells containing β-lactamase. There was a substantial increase in β-lactamase-containing cells when staurosporine pre-treatment was utilized, whereas PGE_2_ did not increase the proportion of β-lactamase-containing cells (Figure 4A). Interestingly, the combination of staurosporine pre-treatment and PGE_2_ during transduction resulted in increased β-lactamase activity compared to vehicle-treated cells, but at lower levels compared to staurosporine treatment alone.

To determine if the combined use of staurosporine and PGE_2_ can increase CD34^+^ transduction, with a higher proportion of transduced cells and more copies of vector per cell, four independent experiments utilizing three unique CD34^+^ cell lots and two different LVV preps were conducted and are summarized in Figure 4B. Cells were treated for 2 hr with varying concentrations (200–800 nM) of staurosporine or vehicle and then transduced in the presence of 10 μM PGE_2_ or vehicle. In all samples, transduction was increased with single-agent use of PGE_2_ or staurosporine. In all samples, combined staurosporine and PGE_2_ increased VCN relative to vehicle-only-treated cells at least as much as either agent alone. In two of four experiments using two unique cell lots, there was an additive effect of combinatorial molecule use (red and blue circles). These data indicate that the pre-treatment of cells with staurosporine and the addition of PGE_2_ to the transduction media can enhance VCN of transduced cells, and in some cases, the combined use of the two molecules will result in further enhanced transduction with no impact on cell viability (data not shown). Staurosporine has a direct impact on the proportion of cells that are transduced, clearly demonstrated in Figure 2B, indicating that increased VCN achieved through combined staurosporine and PGE_2_ usage is at least partially due to increased number of cells with at least one integration and partially due to increased number of integrations per cell.

### Combination Staurosporine and PGE_2_ Treatment Increases Transduction of Human Short-Term NSG Repopulating Cells

**Figure 5:**
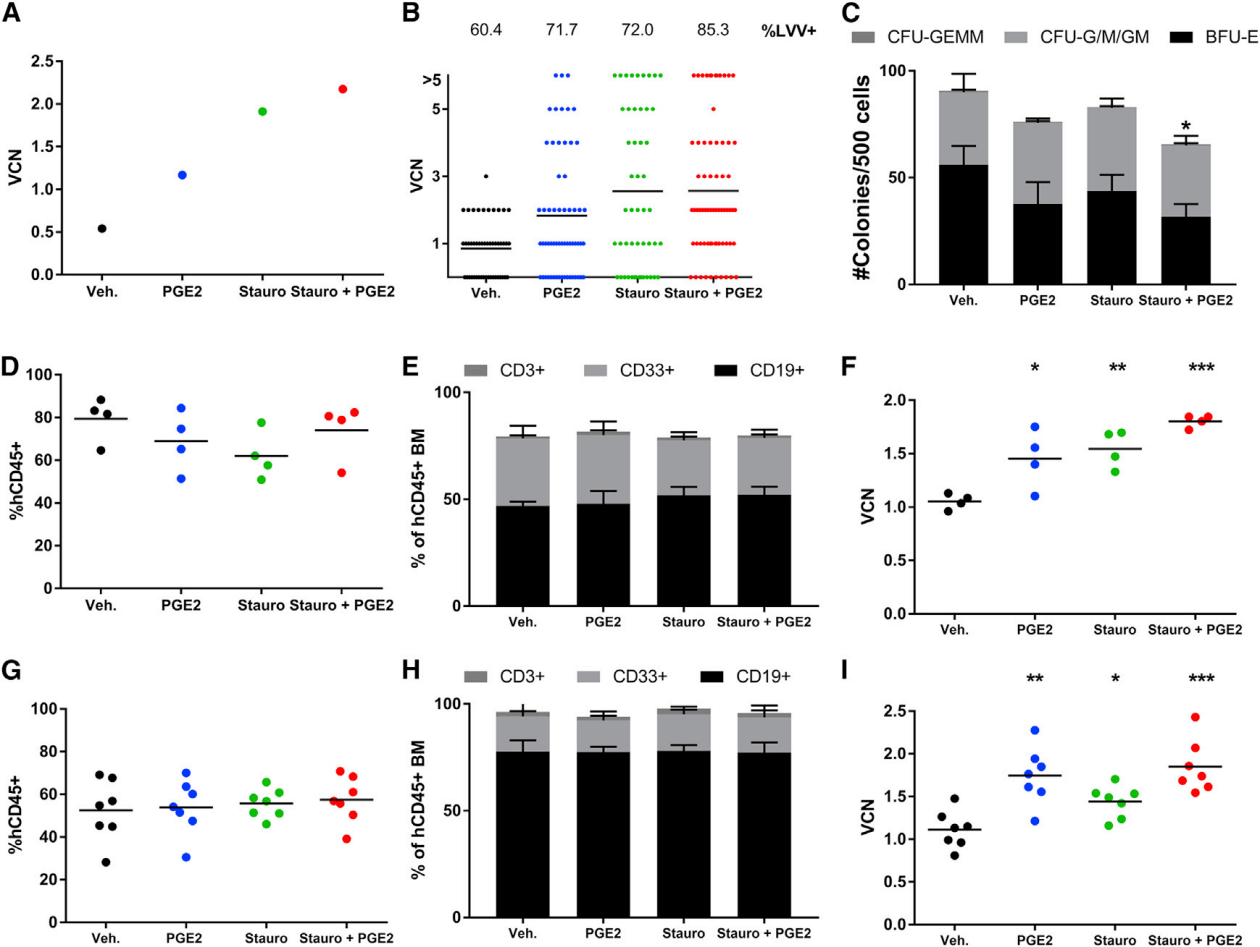
Combination Staurosporine and PGE_2_ Treatment Increases Transduction of Human Short-Term NSG Repopulating Cells (A) VCN analysis of pooled methylcellulose colonies of retained cells (graft) representing what was transplanted into NSG recipients. (B) Individual colony qPCR of graft with %LVV^+^ colonies quantified above each column. (C) Colony enumeration from retained cells plated in methylcellulose. Error bars are 1 SD. (D) Engraftment of human CD45^+^ cells in BM of NSG recipients 2 months post-transplant, n = 5 mice/group. (E) Stacked bar graph depicting myeloid and lymphoid populations present in human CD45^+^ engrafted cells 2 months post-transplant. (F) VCN analysis of BM 2 months post-transplant. (G) Engraftment of human CD45^+^ cells in BM of NSG recipients 4 months post-transplant, n = 10 mice/group. (H) Stacked bar graph depicting myeloid and lymphoid populations present in human CD45^+^ engrafted cells 4 months post-transplant. (I) VCN analysis of BM 4 months post-transplant. Statistical significance is determined relative to vehicle-treated control. *p < 0.05, **p < 0.005, ***p < 0.0005.

To evaluate the potential for a combination of staurosporine and PGE_2_ to increase the transduction of short- and long-term NSG repopulating cells, we selected a concentration of 400 nM staurosporine because engraftment potential was not affected when cells were treated with 400 nM staurosporine, but there was a slight, but not statistically significant, decrease in engraftment when cells were treated with 800 nM staurosporine (Figure 3B). Additionally, when multiple cell lots were examined for transduction after treatment with various concentrations of staurosporine and PGE_2_ present during transduction, there was not a substantial difference in achieved VCNs when comparing the 400 nM and 800 nM staurosporine arms (Figure 4B). Human CD34^+^ cells were treated with vehicle or 400 nM staurosporine for 2 hr and then transduced with BB305 LVV for 24 hr in the presence of vehicle or 10 μM PGE_2_. Cells were transplanted into busulfan-treated NSG mice (1E6 cells per mouse) or further cultured *ex vivo* for analysis of the graft. VCN analysis on pooled methylcellulose colonies (Figure 5A) demonstrated that without any small molecule addition to the transduction media, the VCN of this cell lot was 0.5 vector copies per diploid genome (c/dg). Addition of PGE_2_ increased the VCN 2.4-fold, staurosporine alone increased the VCN 3.8-fold, and combined use of staurosporine and PGE_2_ increased transduction 4.4-fold. In addition to VCN of pooled colonies, individual colonies, representative of single clones, were plucked and analyzed for VCN. As shown in Figure 5B, the addition of staurosporine and PGE_2_, alone or in combination, increased the proportion of LVV^+^ colonies, showing a better transduction efficiency of clonogenic progenitors. Additionally, the VCNs of individual clones were increased when staurosporine or PGE_2_ were added to the transduction media, producing more highly transduced clonogenic progenitors that contribute to the increased median VCN. Importantly, as seen in Figure 5C, there was little impact on the ability of transduced cells to form colonies. Combined use of staurosporine and PGE_2_ resulted in a slight but statistically significant decrease in colony count relative to vehicle-only-treated cells. The plating efficiency, or number of colonies formed relative to input cell number, of vehicle-only-treated cells was 18% and of combination staurosporine and PGE_2_ cells was 13%. In our experience, 10%–20% plating efficiency is typical.

NSG mice were analyzed at both 2 and 4 months post-transplant to look at engraftment of short- and long-term-engrafting HSPCs, respectively. At 2 months post-transplant, there was no significant difference in engraftment as measured by the proportion of human CD45^+^ cells in the bone marrow (BM) of mice treated with cells transduced in the presence of vehicle, PGE_2_, staurosporine, or staurosporine and PGE_2_, as shown in Figure 5D. There was no difference in the *in vivo* differentiation capabilities of the engrafted cells, shown in Figure 5E, with similar proportions of myeloid (CD33^+^) and lymphoid (CD19^+^ or CD3^+^) cells contributing to the human CD45^+^ cellular compartment. Importantly, there was a significant increase in the VCN of engrafted human cells in the BM at 2 months post-transplant (Figure 5F). This was seen when staurosporine or PGE_2_ were used as single agents during the transduction process and the combined use of staurosporine and PGE_2_ further enhanced the VCN of engrafted short-term HSPCs.

Similarly, at 4 months post-transplant, there was no difference in overall engraftment (Figure 5G) or lineage composition of engrafted cells (Figure 5H) when mice are transplanted with cells transduced in the presence of staurosporine, PGE_2_, or staurosporine and PGE_2_. There was a decrease in overall engraftment levels between 2 and 4 months post-transplant, and this decrease is representative of what we typically see. The VCN of engrafted cells in the BM at 4 months post-transplant was significantly increased when staurosporine, PGE_2_, or staurosporine and PGE_2_ were used during transduction compared to vehicle-treated cells. In these long-term engrafting cells, there was no additional benefit of using the combination of staurosporine and PGE_2_ over use of staurosporine or PGE_2_ alone.

## Discussion

Clinical success of LVV-based *ex vivo* gene therapy products will depend at least in part on the ability to efficiently transduce the targeted cell population. Variability in transduction has been seen in both HSPC and T cell genetically modified products, contributing to variable levels of efficacy. Innate cellular restriction pathways responsible for preventing viral infection are significant contributors to this transduction variability. LVV has been shown to trigger some of these pathways and escape others.^22^, ^23^ In diseases where high-level therapeutic transgene expression and/or a high proportion of cells expressing the therapeutic transgene is necessary to achieve clinical efficacy, small molecules added to transduction processes could help overcome innate cellular restriction pathways to LVV transduction.

The serine/threonine protein kinase inhibitor staurosporine has previously been shown to increase infection of HIV in quiescent T cells by stimulating actin dynamics. Spinoculation, a method used to increase transduction efficiency in both HSPCs and T cells, has also been shown to stimulate actin dynamics, suggesting that presence of filamentous actin polymerization during transduction might be a good target for a small-molecule intervention. We have demonstrated here that addition of staurosporine to CD34^+^ cells activates cofilin by preventing an inhibitory phosphorylation resulting in a marked reduction in filamentous actin. These phenotypic changes happen quickly, with a maximum reduction in cofilin phosphorylation evident by 2 hr of incubation with staurosporine. Staurosporine is also a well-studied molecule used to induce apoptosis of cells in a dose-dependent manner and because of this, staurosporine derivatives have been investigated for anti-tumor activity.^24^, ^25^, ^26^ The pro-apoptotic properties of staurosporine caution against prolonged use in HSPCs, and we see evidence of toxicity in HSPCs treated with staurosporine for 24 hr, but not when HSPCs are treated with staurosporine for up to 2 hr. This further supports a strategy of limited staurosporine exposure followed by transduction with LVV.

When CD34^+^ cells are treated with staurosporine prior to transduction with LVV, there is a significant increase in the proportion of cells containing LVV immediately post-transduction, which then leads to an overall increase in the VCN and %LVV^+^ of the transduced population. Cells transduced following staurosporine treatment are polyclonal and, importantly, the addition of staurosporine does not skew the LVV integration profile. The increase in %LVV^+^ cells is seen in both bulk CD34^+^ cells and in a more primitive LT-HSPC population phenotypically defined as CD34^+^CD38LoCD90^+^CD45RA^−^. Further supporting an increase in transduction of true long-term hematopoietic stem cells (HSCs), NSG mice transplanted with CD34^+^ cells treated with staurosporine prior to transduction have a significantly higher VCN in engrafted long-term repopulating cells compared to vehicle-treated control cells. Importantly, there was no difference in the overall engraftment levels or *in vivo* differentiation capabilities of staurosporine-treated cells compared to vehicle-treated or mock-transduced cells.

LVV entry into the cell is one distinct barrier to LVV transduction of HSPCs. Multiple viral restriction pathways exist to prevent infection of HSPCs that could interfere with LVV transduction. Combined use of small molecules known to interfere with unique LVV restriction pathways in a clinical setting could combat patient-to-patient variability from differential activation of restriction-factor pathways. To investigate this, we utilized a combination treatment of staurosporine, to overcome the cellular entry barrier, and PGE_2_, which has been shown to increase LVV transduction efficiency of HSPCs in an entry-independent manner. Treatment of CD34^+^ cells with staurosporine followed by transduction in the presence of PGE_2_ led to increased transduction efficiencies compared to either compound used alone or vehicle controls. Importantly, this combination of molecules does not exhibit signs of overt toxicity *in vitro* via colony formation assay or *in vivo* in a xenogeneic transplant model. NSG mice transplanted with cells treated with both staurosporine and PGE_2_ showed equivalent engraftment levels and myeloid/lymphoid differentiation capabilities as cells treated with single agent or vehicle. This observation was consistent in both short- and long-term NSG repopulating cells. Additionally, in short-term NSG repopulating cells measured 2 months post-transplant, we see significantly higher VCNs when cells are treated with both staurosporine and PGE_2_ during the transduction process compared to vehicle-treated cells and compared to single-agent use prior to or during transduction. While this combinatorial increase is diminished in long-term NSG repopulating cells 4 months post-transplant, there remains a significant increase in VCN with either compound alone or in combination as compared to vehicle-treated cells. Future experiments might include secondary transplantation to further explore the impact of this VCN increase on a more uniform LT-HSC population. Such increases in VCN may be critical in disease indications where high levels of transgene expression are necessary for clinical benefit. More broadly, using combinations of small molecules may also increase the number of patients who can be treated with a given vector lot.

Finally, while the combinatorial strategy employed here did not improve the transduction efficiency of the desired target cell (the LT-HSC) beyond the increases realized with each agent alone, targeting unique LVV cellular restriction pathways remains an interesting strategy for increasing overall transduction efficiencies and decreasing patient-to-patient variability. Overall, these data highlight the potential for the use of small molecules to alleviate LVV transduction barriers in creating genetically modified cellular therapeutics.

## Materials and Methods

### Cell Culture

Research-grade BlaM-containing LVVs were produced at bluebird bio (Cambridge, MA). CD34-enriched mPB samples from healthy human donors were obtained from AllCells (Emeryville, CA) and Key Biologics (Memphis, TN). Research-grade staurosporine was obtained from Millipore Sigma (St. Louis, MO). Research-grade PGE_2_ was obtained from Cayman Chemical (Ann Arbor, MI). CD34^+^ cells were cultured in CellGro stem cell growth media (SCGM; CellGenix, Freiburg, Germany), supplemented with recombinant human cytokines thrombopoietin, Flt3L, and stem cell factor at 100 ng/mL (CellGenix, Freiburg, Germany). CD34^+^ cells were thawed and pre-stimulated for 48 hr at 1E6 cells/mL in cytokine-supplemented media as described above. When applicable, staurosporine was added to the cells at the concentrations and for the length of time indicated in the text. Cells were then collected and washed with SCGM. Cells were transduced with LVV in cytokine-supplemented media as described above, as well as protamine sulfate at a final concentration of 8 μg/mL (APP Pharmaceuticals, Schaumburg, IL) for 24 hr at 4E6 cells/mL. When indicated, 10 μM PGE_2_ was also added to the transduction media. Cells were then washed and maintained in cytokine-supplemented media prior to being subjected to flow cytometric analysis or qPCR for assessment of VCN. Viability post-transduction was assessed via trypan exclusion. For methylcellulose colony-forming unit assays following lentiviral transduction, approximately 500 cells were plated into MethoCult Classic H4434 (StemCell Technologies, Vancouver, BC). Following 12 to 16 days of culture, colonies were scored by morphology, enumerated, and either plucked as individual colonies or pooled and subjected to qPCR for assessment of VCN.

### PCR and VCN Assay

Genomic DNA was isolated from cell cultures using QIAGEN DNeasy protocol (QIAGEN, Hilden, Germany). PCR was performed as described previously.^11^ VCN was assessed relative to an internal reference clone, Clone K3 cDNA, known to contain two copies of integrated viral DNA per diploid genome.^3^

### Assessment of Proportion of LVV-Containing Cells and Colonies

Cells were single-cell sorted into lysis buffer on a 96-well plate. Genomic DNA within cell lysates was amplified with a nested PCR strategy followed by qPCR as above. %LVV^+^ cells is defined as proportion of wells containing signal, defined as having Ct value ≤32, for both the vector and endogenous housekeeping gene out of wells containing signal for the endogenous housekeeping gene. Individual colonies were plucked from methylcellulose into lysis buffer, and stabilization buffer was added per manufacturer’s protocol (TaqMan Sample-to-SNP, Thermo Fisher Scientific, Waltham, MA). Genomic DNA from colony lysates was analyzed via qPCR to determine VCN.

### BlaM Assay

To detect evidence of viral entry into the cell, cells were transduced with LVV containing a β-lactamase fusion protein (Vpr-BlaM). Two hours post-transduction, cells were loaded with fluorescence resonance energy transfer (FRET)-enabled substrate (GeneBLAzer *in vivo* detection kit, Thermo Fisher Scientific, Waltham, MA) for 30–60 min and then washed with CO_2_-independent medium (Gibco, Thermo Fisher Scientific, Waltham, MA). Cells were then plated in CO_2_-independent medium supplemented with 10% fetal bovine serum and left in the dark at room temperature overnight to allow for completion of the enzyme-substrate reaction. The following day cells were analyzed via flow cytometry.

### Engraftment Assay

Female NSG mice were conditioned with 40 mg/kg busulfan 1 day prior to transplantation intravenously with 1E6 CD34^+^ cells. Mice were maintained in sterile conditions and provided with food and water *ad libitum*. At 2 or 4 months post-transplant, bone marrow from the femurs were collected. Cells were analyzed by flow cytometry for human cell-surface markers (CD3, CD19, CD33, and CD45). Additionally, bone marrow was processed for genomic DNA, VCN analysis, and integration site analysis. All protocols were approved by a local institutional animal care and use committee (IACUC; bluebird bio).

### Flow Cytometry

Antibodies were purchased from BioLegend and BD Biosciences. Flow cytometry was performed using an Accuri C6 (BD Biosciences, San Jose, CA), a four-laser special-order research product (SORP) BD Fortessa (BD Biosciences, San Jose, CA), or four-laser SONY SH800 (Sony Biotechnology, San Jose, CA), and analysis was performed using FlowJo software (Tree Star, Ashland, OR). For cell-labeling experiments, sorted cells were incubated in 80 μM CFSE for 5 min.

### Filamentous Actin Staining

Human mPB CD34^+^ cells were cultured for 48 hr in cytokine containing media, described above, and then incubated for indicated periods of time with 800 nM staurosporine. Cells were then fixed, permeabilized, and stained with FITC-labeled phalloidin as in Yoder et al.^13^

### Integration Site Analysis

We followed methods previously described by Zhou et al.^27^ and Tsai et al.^28^ In either method, 1 μg of genomic DNA was sheared using a Covaris sonicator, followed by end repair, A-tailing, and adaptor ligation.

In the linear amplification (LAM)-based method, we carried out the LAM with biotinylated primers as described in Zhou et al.^27^ Cleaned-up LAM products were further amplified using nested PCR with primers carrying sample-specific barcodes.

For the LAM-free assay, we modified the insertion mapping protocol from Tsai et al. by changing PCR primers to the ones listed below in Table 1.^28^ U5 primers contain a sequence found on the U5 part of lentiviral LTR, while P5 and P7 primers provide required Illumina adaptor sequences and barcodes.

**Table 1:** Primer Design for LAM-free LVV Insertion Mapping

First PCR	U5_1	5′-GGATCTCGACGCTCTCCCTACTAGAGATCCCTCAGACCCTTTTAGTC-3′
	P5_1	5′-AATGATACGGCGACCACCGAGATCTA-3′
Second PCR	U5_2	5′-CCTCTCTATGGGCAGTCGGTGATTCCCTCAGACCCTTTTAGTCAGTGTG-3′
	P5_2	5′-AATGATACGGCGACCACCGAGATCTACAC-3′
	P7	5′-CAAGCAGAAGACGGCATACGAGATTCGCCTTAGTGACTGGAGTCCTCTC TATGGGCAGTCGGTGA-3′

In both methods, the libraries were sequenced on Illumina NextSeq in a 150-cycle pair-end run. The reads were trimmed of viral sequences and aligned to hg38 reference genome using bowtie2.^29^ The positions of mapped reads were further annotated with information about corresponding gene models and features taken from the ENSEMBL genomes database.^30^

### Western Blotting

Human mPB CD34^+^ cells were thawed and pre-stimulated at 1E6 cells/mL with 100 ng/mL each stem cell factor (SCF), thrombopoietin (TPO), and Fms-related tyrosine kinase 3 (FLT3) ligand (Flt3L) for 44 ± 4 hr at 37°C, 5% CO_2_. Cells were then treated with 800 nM staurosporine for varying lengths of time. Following treatment, 1E6 cells were collected, washed with 1× PBS, and pelleted. Pellets were stored at −80°C until all time points were collected. Cell pellets were lysed using 300 μL mammalian protein extraction reagent (M-PER) (Thermo Scientific #78501) and protease inhibitors (Thermo Fisher #78430) for 10 min with rocking at room temperature. Cell debris was removed by centrifugation and the collected supernatant was combined with 4× Laemmli loading buffer (Bio-Rad, #161-0747) containing β-mercaptoethanol and heated at 95°C for 3 min. Twenty microliter samples were loaded on a NuPAGE 12% Bis-Tris gels (Invitrogen, #NP0342BOX) and electrophoresed at 200 V for 45 min. The separated proteins were transferred to nitrocellulose membranes using iBlot (novex #IB301001). The membranes were blocked with Odyssey Blocking Buffer (LI-COR Biosciences, #927-40000) and probed for p-cofilin (Santa Cruz, #sc-12912-R at 1:1000) and cofilin (BD Biosciences, #612144 at 1:1000). The bands were visualized using fluorescently tagged secondary antibodies (LI-COR Biosciences, #926-32213 and #926-68070) and imaged on Odyssey CLX using Image Studio 3.1.4 software. Band intensities were measured using ImageJ software.

### Statistical Analysis

Statistical analyses were performed using a two-sided unpaired t test or one-way ANOVA. Statistical significance is indicated with a p value; “ns” denotes p > 0.05.

## Author Contributions

G.L., L.C., J.M., M.L., E.P., Y.S., S.H., O.N., and M.B. performed most of the experiments; G.L., L.C., J.M., Y.S., M.L., O.N., G.V., P.G., and M.B. designed the experiments and analyzed data; M.B. wrote the manuscript, which was reviewed by all authors.

## Conflicts of Interest

The following authors are/were full-time employees of bluebird bio, Inc. and receive salary and hold equity in bluebird bio, Inc.: G.L., L.C., J.M., M.L., E.P., Y.S., S.H., O.N., G.V., P.G., M.B.

## Supplemental Information

Document S1. Figures S1–S3 and Table S1

Document S2. Article plus Supplemental Information
