# Supplementary material for: Staurosporine Increases Lentiviral Vector Transduction Efficiency of Human Hematopoietic Stem and Progenitor Cells
Source: Mol Ther Methods Clin Dev. 2018 Apr 5;9:313–22. doi: 10.1016/j.omtm.2018.04.001 (PMC6054695; doi:10.1016/j.omtm.2018.04.001)
Supplement: Document S1. Figures S1–S3 and Table S1 [file mmc1.pdf]

OMTM, Volume *9*

Supplemental Information

Staurosporine Increases Lentiviral Vector Transduction Efficiency of Human Hematopoietic Stem and Progenitor Cells

Gretchen Lewis, Lauryn Christiansen, Jessica McKenzie, Min Luo, Eli Pasackow, Yegor Smurnyy, Sean Harrington, Philip Gregory, Gabor Veres, Olivier Negre, and Melissa Bonner

Supplemental Figures

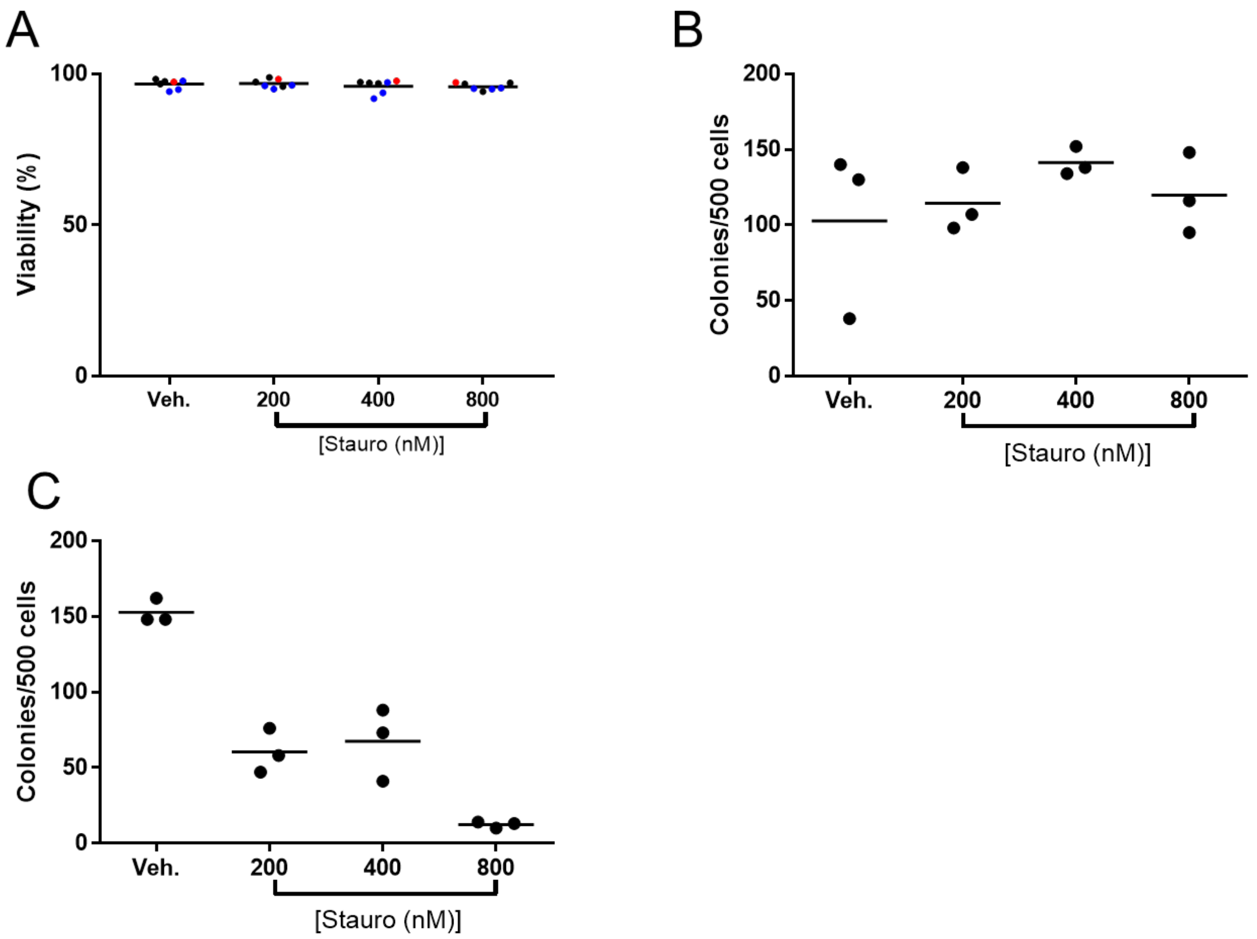

Supplemental Figure 1. Effect of staurosporine on cell survival and progenitor colony forming function. A) Viabilities of cells post-transduction measured by Trypan exclusion. Cells were treated with vehicle or indicated concentration of staurosporine (stauro) for 2 hours prior to transduction. Colors indicate unique experiments. B) Colony enumeration of cells treated with vehicle or indicated concentration of staurosporine for 2 hours, transduced, and plated in methylcellulose. C) Colony enumeration of cells treated with vehicle or indicated concentration of staurosporine for 24 hours, transduced, and plated in methylcellulose.

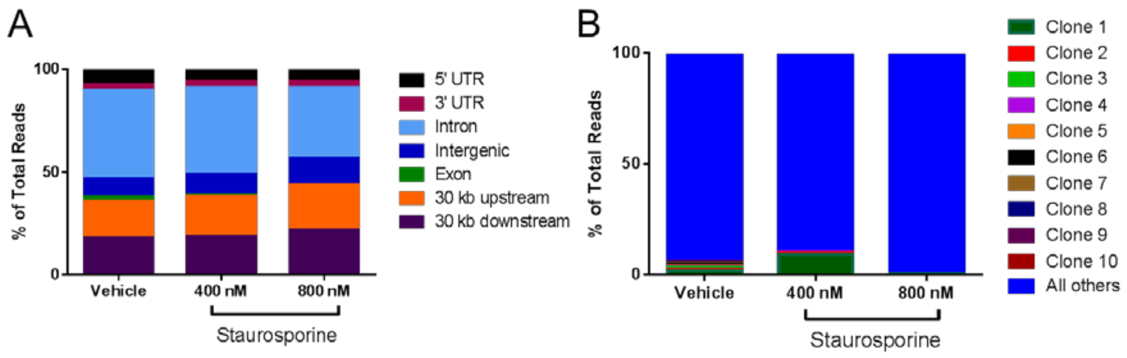

Supplemental Figure 2. ISA analysis of graft. A) Retained cells pre-transplant were analyzed for integration site within gene bodies. B) Retained cells pre-transplant were analyzed for integration sites. Top 10 clones captured via ISA are shown, note that Clones 1-10 represent different integration sites within each transplant arm.

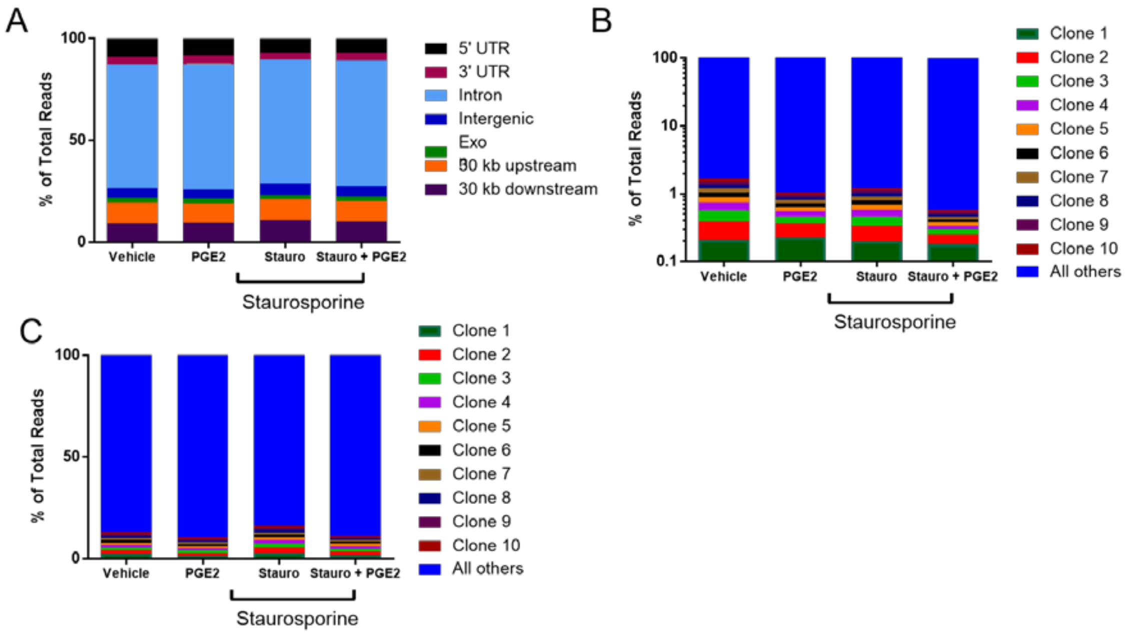

Supplemental Figure 3. ISA analysis pre- and post-transplant. A) Retained cells pre-transplant were analyzed for integration site within gene bodies. B) Retained cells pre-transplant were analyzed for integration sites. Top 10 clones captured via ISA are shown. C) Top 10 integration sites from pooled BM 4 months post-transplant. Note that Clones 1-10 represent different integration sites within each transplant arm and are different between B and C.

Supplemental Table 1. VCN fold differences compared to vehicle treated cells in prospectively identified low, mid, and high transducing cell lots treated with indicated concentrations of staurosporine for 2 hours prior to transduction. Color of cell indicates rank order (green>yellow>red) within table with green indicating highest fold increase in VCN and red lowest fold increase in VCN.

	Low	Mid	High
200 nM	1.90	1.30	1.57
400 nM	2.03	1.63	1.40
800 nM	2.36	1.61	1.28
